# An estimate of rate of deviation from NCCN guideline recommendations for central nervous system imaging in trials forming basis for drug approval in first line advanced non-small cell lung cancer (NSCLC)

**DOI:** 10.1186/s12885-022-09179-y

**Published:** 2022-01-16

**Authors:** John Sharp, Vinay Prasad

**Affiliations:** 1 Department of Medicine, UCLA Health, Los Angeles, California USA; 2 Department of Medicine, University of California San Francisco, 550 16th St., San Francisco, California CA 94158 USA; 3 Department of Epidemiology and Biostatistics, University of California San Francisco, 550 16th St., San Francisco, California CA 94158 USA

## Abstract

**Importance:**

It is unknown whether and to what degree trials submitted to the US FDA to support drug approval adhere to NCCN guideline-recommended care in their baseline and surveillance CNS imaging protocols.

**Objective:**

We sought to characterize the frequency with which the trials cited in US FDA drug approvals for first line advanced NSCLC between 2015 and 2020 deviated from NCCN guideline-recommended care for baseline and surveillance CNS imaging.

**Design, setting, and participants:**

Retrospective observational analysis using publicly available data of (1) list of trials cited by the FDA in drug approvals for first line advanced NSCLC from 2015 to 2020 (2) individual trial protocols (3) published trial data and supplementary appendices (4) archived versions of the NCCN guidelines for NSCLC from 2009 to 2018 (the years during which the trials were enrolling).

**Main outcomes and measures:**

Estimated percentage of trials for first line advanced NSCLC leading to FDA approval which deviated from NCCN guideline-recommended care with regards to CNS baseline and surveillance imaging.

**Results:**

A total of 14 studies that had been cited in FDA drug approvals for first line advanced NSCLC met our inclusion criteria between January 1, 2015 and September 30, 2020. Of these trials, 8 (57.1%) deviated from NCCN guidelines in their baseline CNS imaging requirement. The frequency of re-assessment of CNS disease was variable amongst trials as well, with 9 (64.3%) deviating from NCCN recommendations.

**Conclusions and relevance:**

The trials supporting US FDA drug approvals in first line advanced NSCLC often have CNS imaging requirements that do not adhere to NCCN guidelines. Many trials permit alternative, substandard methods and the proportion of patients undergoing each modality is uniformly not reported. Nonstandard CNS surveillance protocols are common. To best serve patients with advanced NSCLC in the US, drug approvals by the FDA must be based on trials that mirror clinical practice and have imaging requirements consistent with current US standard of care.

## Key points

Question How many drugs for first line advanced non-small cell lung cancer (NSCLC) received US Food and Drug Administration (FDA) approval on the basis of trials that deviated from National Comprehensive Cancer Network (NCCN) guideline recommendations regarding baseline and surveillance central nervous system (CNS) imaging?

Findings In this retrospective observational study among 14 trials for drugs used in first line advanced NSCLC that formed the basis for FDA approval between 2015 and 2020, 8 (57.1%) deviated from NCCN guideline-recommended care for baseline CNS imaging. 9 (64.3%) trials had CNS surveillance imaging protocols that deviated from NCCN guideline-recommended care.

Meaning Many of the trials that led to US FDA approval of drugs in first line advanced NSCLC relied on baseline and surveillance CNS imaging protocols that deviated from NCCN guideline-recommended care. When discrepancies in baseline and surveillance CNS imaging exist between clinical trial protocols and real world practice, the generalizability of benefits observed in the trial population becomes less certain.

## Introduction

Over 228,000 people will be diagnosed with lung cancer each year in the United States and the vast majority will present with non-small cell lung cancer (NSCLC) [1] . Lung cancer is the leading cause of cancer-related mortality in the United States and 5-year survival rates remain below 25%. A 1995 BMJ meta-analysis of 8 clinical trials comparing platinum-based chemotherapy to best supportive care demonstrated the most effective chemotherapy regimens of the day increased the one-year survival rate from 5 to 15% [2] . New therapeutic options have improved patient outcomes, with the average one-year survival for advanced forms of the disease now exceeding 25% [1] . Continued improvement in patient outcomes relies upon well-designed clinical trials comparing novel therapeutic agents to the current best standard of care. Unfortunately, trials are not always designed in this fashion. Many trials in oncology utilize control arms that are disputed and potentially inferior to other available options [3, 4] . A reliance on surrogate endpoints and the inappropriate use of post-protocol therapies (or crossover) have also been cited as deficiencies in pivotal registration studies submitted to the US FDA [5] .

However, what remains unknown is whether imaging – at baseline and to monitor disease – in these studies mirrors US standards of care. Inadequate staging at baseline, particularly of the CNS, may result in occult intracranial disease going untreated, which has both prognostic and therapeutic implications. Frequent follow up imaging of the CNS may artificially demonstrate gains in progression, though these may not be recapitulated in clinical settings where frequent surveillance CNS imaging is nonstandard. For these reasons, we sought to investigate the use of CNS imaging in clinical trials of drugs approved by the US FDA to treat advanced NSCLC and how this differs from the standard of care in US clinical practice. The National Comprehensive Cancer Network (NCCN) guidelines, derived from clinical trial data and expert consensus, are used by as many as 95% of US oncologists [6] and are now Medicare compendia, which mandates that Medicare base payment decisions on them and thus codifies these guidelines as a US standard bearer.

## Methods

### Overview

Our study was not submitted for institutional review board approval because it did not involve healthcare records and all data are publicly available. The study was conducted between September 23, 2020 to December 8, 2020. We sought to estimate the percentage of clinical trials leading to FDA drug approval for first line advanced NSCLC that had protocols which deviated from NCCN guideline recommendations, with regards to CNS baseline and surveillance imaging. We examined all FDA approvals for first line advanced NSCLC between January 1, 2015 and September 23, 2020. We then considered only phase 3 or combined phase 2/3 trials that were cited in FDA announcements as the basis for drug approvals in the first line advanced NSCLC space. We analyzed only those studies with readily available protocol documents to determine the nature of the baseline and surveillance CNS imaging within the trial. We compared these protocols to the archived versions of NCCN guidelines for advanced NSCLC that would have been relevant during enrollment of these trials. We report on the findings of the protocols’ deviation from NCCN guidelines.

### Data set

#### Study selection

We examined all US FDA oncology drug approvals for first line advanced NSCLC from January 1, 2015 through September 23, 2020 based on data available at the FDA website. We included phase 3 and combined phase 2/3 trials with a standard-of-care comparator arm that had publicly available complete protocol documents. We retrieved the published manuscripts of the clinical trials cited as the basis for each approval as well as their supplementary appendices and protocols. We did not consider trials that had not yet been published at the time of investigation (CHECKMATE 9LA). We also did not consider phase 1, phase 2, latter line phase 3 trials or accelerated approvals.

#### Data extracted

For each cited trial, we catalogued the dates of active enrollment of subjects, stages of subjects enrolled, name of the drug/combination therapy receiving approval on the basis of the trial, comparator treatment arm, date of approval, specific treatment indication, whether the drug was a new molecular entity, whether the trial was multinational, the continents on which the trial was conducted, whether brain MRI was required at baseline, alternative accepted CNS imaging modalities within the trial, and the schedule of assessment of CNS disease. Additionally, in cases where brain MRI was not mandated for CNS staging in all subjects, we examined data reported in the trial manuscripts and supplements for information on the frequency with which alternative CNS assessments were performed. Archived versions of the NCCN guidelines for NSCLC were reviewed for their recommendations regarding baseline and surveillance CNS imaging and these were compared to the protocols of the trials considered.

#### Determining NCCN guideline recommendations

Archived versions of the NCCN guidelines for NSCLC were obtained with the express consent of the NCCN. These guidelines had been published from November 30, 2009 to August 17, 2018, which encompassed the entire enrollment periods of all trials under consideration. As many versions of these guidelines are published each year, each individual version of the guidelines was reviewed for its recommendations.

To determine the recommendations for baseline CNS imaging, the Evaluation and Treatment sections of the NCCN guidelines for stages IIIA, IIIB, and IV were reviewed. Because these trials only considered advanced NSCLC, the recommendations for stages less than IIIA were not reviewed. Each version of the guidelines was categorized by its recommendation of baseline brain MRI as being either 1) indicated or 2) not indicated. The recommendations from the versions of the NCCN guidelines encompassing each trial’s enrollment period were then used to compare the contemporaneous NCCN guideline-recommended care to each trial protocol.

To determine the recommendations for surveillance CNS imaging, the Surveillance section of the NCCN guidelines were reviewed. The surveillance recommendations for all stages of NSCLC, ranging from no evidence of disease to stage IV were reviewed to encompass the recommended reassessments for all potential degree of response subgroups within each trial. For each version of the NCCN guidelines, the surveillance recommendation was categorized as 1) brain MRI routinely indicated or 2) brain MRI not routinely indicated. The recommendations from the versions of the NCCN guidelines encompassing each trial’s enrollment period were then used to compare the contemporaneous NCCN guideline-recommended care to each trial protocol.

#### Determining baseline CNS imaging requirements

To determine the number of trial protocols deviating from NCCN guidelines for baseline CNS imaging, all trial protocols were reviewed for their baseline CNS imaging requirements. Methods sections, trial flow charts, and schedule of assessments tables were reviewed to determine how CNS imaging was performed at baseline. Protocols were then compared to contemporaneous NCCN guidelines and categorized as either 1) adhering to NCCN guidelines for CNS imaging at baseline for all subjects, 2) not adhering to NCCN guidelines for CNS imaging at baseline for all subjects or 3) unclear. For protocols falling into the second category, a text description of CNS imaging requirements was collected. We then divided the number of trials that deviated from NCCN guidelines for baseline CNS imaging by the total number of trials considered to determine the percentage of trials not meeting NCCN guideline recommendations for CNS imaging.

#### Determining reporting of CNS imaging modality

All protocols were reviewed for CNS imaging requirements as described above. For protocols that did not adhere to NCCN guidelines for baseline CNS imaging for all subjects, text descriptions of alternative baseline CNS imaging modalities and practices, as described in the Methods sections, were recorded. The published trials and their supplementary appendices were then reviewed for reporting on the frequency with which alternative CNS imaging modalities and practices were performed. Where available, these values were recorded.

#### Determining CNS surveillance imaging requirements

The methods sections, trial flow charts, and schedule of assessments tables of all trial protocols were reviewed to determine the frequency with which CNS reassessment was required. The protocols were categorized as 1) mandating CNS reassessment with each scheduled assessment of disease for all subjects, 2) mandating CNS reassessment with each scheduled assessment of disease for subjects with known baseline CNS metastatic disease, 3) CNS reassessment as clinically indicated or 4) unclear. We calculated the percentage of trials within each category by dividing the number of trials within each category by the total number of trials considered.

We further compared each trial protocol’s schedule for CNS reassessment to the schedule for CNS reassessment recommended by the NCCN guidelines for the dates corresponding to the enrollment period for each individual trial. Because the NCCN recommendation for CNS reassessment did not vary by burden of disease (i.e no evidence of disease vs stage IV), subgroup analysis by treatment response was not performed. We then calculated the percentage of trials deviating from NCCN guideline recommended CNS surveillance imaging by dividing the number of trials with protocols that did not adhere to the contemporaneous NCCN guidelines by the total number of trials considered.

### Statistical analysis

We sought to provide a descriptive estimate of the percentage of trials leading to FDA drug approval for advanced NSCLC that deviated from NCCN guideline-recommended care for baseline and surveillance CNS imaging. Analysis was performed using Microsoft Excel.

## Results

**Fig. 1:**
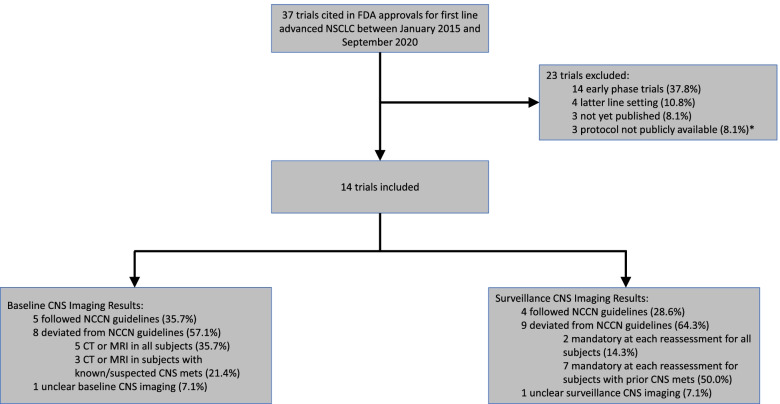
Flowchart of Trials Forming Basis for FDA Approval for first Line Advanced NSCLC Therapeutics from 2015 to 2020. *Two trials were both early phase and not yet published

We examined 37 FDA approvals for first-line treatment of advanced NSCLC between January 1, 2015 and September 23, 2020. Of these, 14 (37.8%) met our inclusion criteria, as seen in Fig. 1. The main reasons for exclusion were pivotal data emerged from a phase 1 or 2 trial (*n* = 14), latter line setting (*n* = 4), trial was not yet published (*n* = 3), and protocol was not publicly available (*n* = 3). Two trials were both early phase trials and unpublished.

### Results of NCCN guideline audit

Forty versions of the NCCN guidelines were published between November 30, 2009 to August 17, 2018. For stage IIIA, IIIB, and IV NSCLC, the NCCN recommended MRI brain with contrast as baseline CNS imaging in 40 (100%) of the versions. For stage IIIA, IIIB, and IV NSCLC, the NCCN stated that surveillance brain MRI is not routinely indicated in 40 (100%) of the versions.

### Baseline CNS imaging assessments

As seen in Table 1, of the 14 trials we considered, 8 (57.1%) deviated from NCCN guidelines with respect to baseline CNS imaging requirements. 5 (35.7%) allowed either CT or MR but did require baseline CNS imaging in all participants. 3 (21.4%) mandated some form of baseline CNS imaging, either MRI or CT, in all subjects with a known or suspected history of CNS metastases. Only 5 (35.7%) explicitly mandated baseline brain MRI in all trial participants as recommended by the NCCN. 1 (7.1%) did not have explicit baseline CNS imaging requirements elaborated in their protocols.

**Table 1:** Summary of trials cited as basis for FDA approval for drugs used in first line advanced NSCLC and their baseline and surveillance CNS imaging requirements

Trial Name	Drug and Indication	NCT	Baseline CNS Imaging Required	Surveillance CNS Imaging Required
RELAY	Ramucirumab + Erlotinib, first line EGFR (19del, L858R)	NCT02411448	MRI brain required for all subjects	As clinically indicated
ALTA-1 L	Brigatinib, first line ALK mutated	NCT02737501	MRI brain required for all subjects	At regular imaging intervals in all subjects
Impower110	Atezolizumab, first line PDL1 high	NCT02409342	CT or MRI brain required for all subjects	At regular imaging intervals in subjects with CNS disease
CHECKMATE-227	Ipilimumab + Nivolumab, first line PDL1 > 1%	NCT02477826	MRI brain required for all subjects	At regular imaging intervals in subjects with CNS disease
IMpower130	Atezolizumab with carboplatin/protein-bound paclitaxel, first line non-squamous	NCT02367781	CT or MRI brain required for all subjects	As clinically indicated
KEYNOTE-042	Pembrolizumab, first line PDL1 > 1%	NCT02220894	Unclear*	Unclear*
KEYNOTE-407	Pembrolizumab + carboplatin/paclitaxel, first line	NCT02775435	CT or MRI brain accepted**	At regular imaging intervals in subjects with CNS disease
ARCHER 1050	Dacomitinib, first line EGFR (19del, L858R)	NCT01774721	CT or MRI brain required for all subjects	As clinically indicated
KEYNOTE-189	Pembrolizumab + pemetrexed/carboplatin, first line	NCT02578680	CT or MRI brain accepted**	At regular imaging intervals in subjects with CNS disease
FLAURA	Osimertinib, first line EGFR (19del, L858R)	NCT02296125	CT or MRI required if known/suspected CNS disease	At regular imaging intervals in subjects with CNS disease
ASCEND-4	Ceritinib, first line ALK mutated	NCT01828099	CT or MRI brain required for all subjects	At regular imaging intervals in subjects with CNS disease
ALEX	Alectinib, first line ALK mutated	NCT02075840	MRI brain required for all subjects	At regular imaging intervals in all subjects
KEYNOTE-024	Pembrolizumab, first line PDL1 high	NCT02142738	MRI brain required for all subjects	As clinically indicated
SQUIRE	Necitumumab + gemcitabine/cisplatin, first line squamous	NCT00981058	CT or MRI brain required for all subjects	At regular imaging intervals in subjects with CNS disease

*Protocol, supplement, and manuscript do not explicitly discuss requirement

**Protocol is not explicit if all subjects or only subjects with history of CNS disease underwent baseline CNS screening

Of the 9 (64.3%) studies that did not explicitly mandate NCCN guideline-recommended baseline CNS imaging (including the 1 study with unclear requirements), 0 (0%) reported the frequency with which alternative modalities, such as CT brain, were used.

### CNS surveillance strategies

Of the 14 trials considered, 9 (64.3%) deviated from NCCN recommended CNS surveillance strategies. 2 (14.3%) mandated CNS imaging at each scheduled disease assessment in all patients. 7 (50.0%) mandated CNS imaging at each scheduled disease assessment in all patients with a known history of CNS disease at baseline. Four trials (28.6%) specified that repeat CNS assessment should occur as clinically indicated, consistent with NCCN guidelines. 1 (7.1%) had unclear CNS surveillance protocols.

### Multinational trials

All 14 (100%) trials that were examined occurred in the multinational setting. 2 (14.3%) trials enrolled no patients in the United States at all.

### New molecular entities

The 14 trials considered evaluated 9 different drugs. Of the 9 drugs, 3 (33.3%) were new molecular entities.

## Discussion

Clinical trials supporting US Food and Drug Administration approval for advanced NSCLC therapies should ideally assess efficacy of novel therapies over available standard of care in the United States. When the care provided in a trial setting deviates significantly from clinical practice, the conclusions of the trial are not as generalizable to real world patients. Prior analyses have examined deviations from standard of care in the form of deficiencies in control arm choice and post-protocol therapies; however, our study is the first to examine the role of intracranial imaging [3–5] .

We find that 57.1% of trials do not adhere to NCCN guideline recommendations for baseline CNS imaging, either by accepting CT brain, which is less sensitive than the US standard of MRI, or by failing to screen all subjects for CNS disease, or both. 35.7% performed baseline CNS imaging adherent to NCCN guidelines. 7.1% had unclear baseline CNS imaging requirements. The frequency with which substandard imaging was employed was universally not reported. After initiation of therapy, 64.3% performed surveillance CNS imaging in a manner discordant with NCCN guideline recommendations, 28.6% performed CNS imaging as appropriately indicated, and 7.1% had unclear CNS surveillance imaging protocols.

To our knowledge, this is the first investigation to report on baseline and surveillance imaging protocols in trials leading to drug approvals in first line advanced NSCLC. The findings raise important questions regarding both the care provided to patients within the trials as well as the generalizability of the benefits reported in the trial setting to patients with advanced NSCLC receiving treatment in the US.

First, deviations from NCCN guidelines regarding baseline CNS imaging have the potential to “down-stage” patients at baseline. MRI has been the standard NCCN recommended imaging modality to assess for CNS disease since at least 2009, the earliest any of the trials considered began enrollment. This is due to its superior sensitivity compared to CT [7, 8] , particularly for metastases less than 5 mm in size, in addition to its lack of ionizing radiation and superior tissue resolution. By accepting CT brain studies in place of MRI brain with contrast, as is recommended by the NCCN in the workup of all NSCLC patients with advanced disease (i.e. stages IIIA and above), CNS metastases present at baseline, particularly small ones, may be missed initially but subsequently scored as progression of disease when detected on surveillance imaging. If a trial is assessing an adjuvant therapy, lack of appropriate brain imaging may miss occult metastatic disease. Substandard baseline CNS imaging raises important ethical questions as it would be beneath the US standard of care to deprive patients with CNS disease appropriate radiation therapy. Additionally, significant differences with respect to prognosis have been described [9] between patients with CNS metastases at baseline and those without. In trials accepting alternative CNS imaging modalities, imbalances in the proportion of patients undergoing baseline brain MRI between treatment arms could contribute to differences in outcomes related to presence of occult CNS disease rather than the effect of the therapeutic intervention. It is difficult to interpret how much of an impact these practices have on the overall results of many of these trials as none report the proportion of subjects undergoing each modality of baseline CNS screening.

The other deviation from NCCN guideline-recommended care we observed is that many trials only required baseline CNS imaging in patients with a known or suspected history of CNS disease. While it is possible that diagnostic imaging consistent with the local standard of care was obtained prior to trial enrollment for these patients, it cannot be assumed that local standard of care was consistent with the US standard of care due to the widely multinational nature of these trials. Brain metastases are estimated to occur in 41% of patients with NSCLC [10] and by accepting head CT in place of the diagnostically superior brain MRI, which is the standard of care in the US, it is highly plausible that a proportion of patients in these trials had undetected baseline CNS disease. Because the trials do not report the proportion of patients in each arm undergoing each CNS imaging modality, the effect on the results is uncertain. Regardless, one way to be more confident that clinical benefits observed in trials will translate to patients in US oncology clinics is for the FDA to ensure that trials that form the basis for drug approval have protocols that adhere to US standard of care with regards to their imaging.

Second, deviations in CNS surveillance imaging from NCCN guidelines create confusion regarding a drug’s benefit. This is particularly relevant in the case of novel agents targeting the ALK pathway (alectinib, brigatinib), which have been touted specifically for their superior CNS penetration. By mandating CNS reassessments at each scheduled imaging interval, as was done in trials investigating these agents, a perceived benefit of better disease control within the CNS was observed. Indeed, a lower cumulative incidence of CNS progression and a superior CNS objective response rate, respectively, were reported as secondary outcomes in these trial manuscripts [11, 12] . However, this is not what is routinely done in real world Oncology clinics. It remains unclear whether this radiologic benefit observed in the setting of serial asymptomatic imaging is necessarily one that translates into a clinical benefit when imaging occurs in response to symptoms, as it does in NCCN guideline-directed care.

Third, all trials we examined occurred in the multinational setting, with two trials enrolling no patients at all in the United States, yet they have formed the basis for approval by the FDA for use in the United States [13–24]. While this is not inherently problematic, as well done randomized controlled trials can be performed in many countries, if the care provided in the trial is below the standard of care in the United States, this creates problems with the generalizability of any observed benefit to patients in the United States. It may be that accepting alternate CNS imaging modalities such as CT is a practical necessity based on local availability, but this is not acceptable care for patients with advanced NSCLC in the US. Imbalances in the proportion of patients undergoing brain MRI between treatment arms could represent a potential confounder as patients with undetected CNS disease have an overall worse prognosis a priori compared to those without CNS disease. Therefore, the FDA must interpret trials which implement alternative means of CNS disease assessment with the utmost caution.

### Limitations

A number of limitations to our study must be acknowledged. First, this study looked specifically at the space of advanced NSCLC. The particular pattern of deviation from US standards of care may be unique to this space.

Second, as we only examined trials with publicly available protocols and supplemental appendices, it is possible that we overestimate the number of trials deviating from current US standard of care. We excluded three trials due to their protocols not being readily accessible and designated two included trials as having “unclear” imaging and surveillance protocols and it is possible that the practices in these trials was entirely consistent with US standard practices.

Third, we only examined drugs granted approval after phase III trials in which comparison to a control group is undertaken. As many drugs received accelerated approval on the basis of phase I and II trials, it is possible that the protocols of these trials adhered to US standards with regard to baseline and surveillance imaging. We excluded 14 trials due to their phase I or II nature.

Fourth, it must be stated that it is possible, even likely, that the data regarding the frequency of alternate baseline CNS imaging modality use exists. We did not submit requests for additional data from the trials in which either baseline CT or MRI were permissible. For the purposes of this investigation, the fact that these data are not publicly available is what renders these trials difficult to completely interpret. When alternate modes of CNS imaging besides that recommended by the most up to date guidelines are used, it should be reported in the trial, or at least in the supplementary materials.

## Conclusion

While great strides forward have been made with regard to therapeutics for advanced NSCLC, continued progress depends upon well-designed clinical trials that rigorously test novel agents against existing standards of care. Trial protocols that fall below the current US standard of care have the potential to create confusion about a new therapeutic agent’s true benefit by “downstaging” subjects at baseline, missing known metastatic disease, introducing confounders if imbalances exist in imaging techniques between treatment arms, as well as detecting asymptomatic CNS progression that might not have been detected in real world settings. Additionally, it raises ethical concerns about the adequacy of care provided for patients within the trials as CNS disease may be going undetected and undertreated. Nevertheless, these trials are cited as the basis for new drug approvals by the FDA. We found that 57.1% of trials in first line advanced NSCLC did not strictly adhere to NCCN recommended baseline CNS imaging and that, when alternatives were used, the alternate modality was not reported. Additionally, 64.3% of trials were not strictly adherent to NCCN recommendations for CNS surveillance strategies. These somewhat subtle deviations from current US standard of care for advanced NSCLC have the potential to create confusion regarding a drug’s true benefit. In order to better serve patients with advanced NSCLC in the US, trials which form the basis for drug approvals by the FDA must adhere to current best practices which involves adherence to standard CNS baseline imaging and surveillance protocols.

## Data Availability

The datasets used and/or analysed during the current study are available from the corresponding author on reasonable request.

## Abbreviations

NSCLC: Non-small cell lung cancer
FDA: Food and Drug Administration
NCCN: National Comprehensive Cancer Network
CNS: Central Nervous System
CT: Computerized Tomography
MRI: Magnetic Resonance Imaging
